# CX-4945 and siRNA-Mediated Knockdown of CK2 Improves Cisplatin Response in HPV(+) and HPV(−) HNSCC Cell Lines

**DOI:** 10.3390/biomedicines9050571

**Published:** 2021-05-18

**Authors:** Janeen H. Trembley, Bin Li, Betsy T. Kren, Amy A. Gravely, Emiro Caicedo-Granados, Mark A. Klein, Khalil Ahmed

**Affiliations:** 1 Minneapolis VA Health Care System Research Service, Minneapolis, MN 55417, USA; krenx001@umn.edu (B.T.K.); amy.gravely@va.gov (A.A.G.); ahmedk@umn.edu (K.A.); 2 Department of Laboratory Medicine and Pathology, University of Minnesota, Minneapolis, MN 55455, USA; 3 Masonic Cancer Center, University of Minnesota, Minneapolis, MN 55455, USA; Emiro.Caicedo-Granados@va.gov (E.C.-G.); mark.klein2@va.gov (M.A.K.); 4 Minneapolis VA Health Care System Otolaryngology Section, Minneapolis, MN 55417, USA; bin.x.li@kp.org; 5 Department of Otolaryngology, University of Minnesota, Minneapolis, MN 55455, USA; 6 Minneapolis VA Health Care System Hematology and Oncology Section, Minneapolis, MN 55455, USA; 7 Department of Medicine, University of Minnesota, One Veterans Drive, Minneapolis, MN 55417, USA

**Keywords:** head and neck cancer, HNSCC, human papillomavirus, HPV, CK2, NFκB, cisplatin, PDCD4, p21

## Abstract

Head and neck squamous cell carcinoma (HNSCC) can be categorized into human papillomavirus (HPV) positive or negative disease. Elevated protein kinase CK2 level and activity have been historically observed in HNSCC cells. Previous studies on CK2 in HNSCC did not generally include consideration of HPV(+) and HPV(−) status. Here, we investigated the response of HPV(+) and HPV(−) HNSCC cells to CK2 targeting using CX-4945 or siRNA downregulation combined with cisplatin treatment. HNSCC cell lines were examined for CK2 expression levels and activity and response to CX-4945, with and without cisplatin. CK2 levels and NFκB p65-related activity were high in HPV(+) HNSCC cells relative to HPV(−) HNSCC cells. Treatment with CX-4945 decreased viability and cisplatin IC50 in all cell lines. Targeting of CK2 increased tumor suppressor protein levels for p21 and PDCD4 in most instances. Further study is needed to understand the role of CK2 in HPV(+) and HPV(−) HNSCC and to determine how incorporation of the CK2-targeted inhibitor CX-4945 could improve cisplatin response in HNSCC.

## 1. Introduction

Investigations over the past half century have shown that protein kinase CK2 is a multifaceted master regulator of cell function in both normal and disease states [1,2,3,4]. CK2 has enzymatic activity as a monomer (CK2α or CK2α’) or as a tetramer with two α and/or α’ catalytic subunits linked via two β subunits, which serve a regulatory role. This highly conserved kinase phosphorylates primarily serine and threonine amino acids. Phosphosites for CK2 number close to one thousand (phosphositeplus website [5]) and these substrate proteins are found in nuclear and cytoplasmic compartments, organelles, and subdomains; thus, the abundance of CK2 targets underpins its involvement in a large number of cellular activities [6]. In the context of cancer cells, CK2 functions in a global sense to promote cell growth and proliferation and suppress apoptosis [7]. In the majority of cancers, increased CK2 activity and expression relative to normal tissue is well documented [8,9]. In head and neck squamous cell carcinoma (HNSCC), data indicate that elevated CK2 is associated with aggressive tumor behavior and poor clinical outcome [9,10,11].

Human papillomavirus infection is a causative agent for HNSCC, and HNSCC is currently categorized into HPV(+) or HPV(−) disease in part due to differential survival prognoses [12,13,14,15,16]. CK2 is known to phosphorylate numerous viral proteins [17]. In the case of HPV, CK2 substrates include the replication factor E1 and the transforming protein E7, which inactivates the tumor suppressor pRb [18,19,20,21,22]. In addition, CK2 phosphorylation of the chromatin-associated bromodomain-containing protein 4 (Brd4) influences HPV and cellular transcription [23,24].

Given CK2 influence over the HPV lifecycle and the general lack of data on the efficacy of targeting CK2 in HPV(+) HNSCC, we investigated the potential effect of reducing CK2 activity or protein levels on the viability of HPV(+) vs. HPV(−) HNSCC cell lines. Further, due to the common use of cisplatin in HNSCC patient therapy, we also examined the response of multiple HPV(+) and HPV(+) cell lines to combined CK2 targeting and cisplatin treatment. Our data demonstrated that CK2 expression levels and NFκB p65-directed phosphorylation are higher in HPV(+) compared to HPV(−) HNSCC. Further, inhibition of CK2 with CX-4945 (Silmitasertib) effectively reduced the viability of both types of HNSCC cell lines, decreased the IC50 for cisplatin in all cell lines, and demonstrated synergy with cisplatin in 2 cell lines. Our results suggest that, despite additional roles for CK2 in the biology of HPV(+) HNSCC, blocking CK2 activity has good potential as a therapeutic strategy to improve cisplatin response in HNSCC.

## 2. Materials and Methods

### 2.1. Cell Lines, Culture, and Drugs

The cell lines UM-SCC-47 and 93-Vu-147T were from the stocks of Dr. Emiro Caicedo-Granados (UM-SCC-47 originally from Dr. Thomas Carey, University of Michigan, USA; 93-Vu-147T originally from Dr. John Lee, previously Sanford Health, South Dakota, USA; now Avera Cancer Institute, South Dakota, USA). UPCI-SCC-90 cells were obtained from Dr. John Lee (Avera Cancer Institute, South Dakota, USA). UM-SCC-6 cells were purchased from EMD Millipore (Temecula, CA, USA). UM-SCC-47, UM-SCC-6, 93-Vu-147T, and UPCI-SCC-90 sells were cultured in Hyclone Dulbecco’s modified Eagle’s medium—high glucose with sodium pyruvate (SH30243.01, Cytiva Lifesciences, Marlborough, MA, USA), 10% fetal bovine serum (FBS, Atlanta Biologicals, R&D Systems, Minneapolis, MN, USA), 1X non-essential amino acids (Specialty Media, ThermoFisher Scientific, Waltham, MA, USA), and 1% penicillin/streptomycin (ThermoFisher Scientific, Waltham, MA, USA). Detroit-562 and Fadu cells were purchased from ATCC (Manassas, VA, USA) and grown in Eagle’s minimum essential medium (SH30024.01, GE Healthcare, Chicago, IL, USA) with 10% FBS and 1% penicillin/streptomycin. Human epidermal keratinocytes (HEKn pooled) were purchased from ThermoFisher Scientific (A13401) and grown as recommended by the manufacturer. All cell lines were grown in an incubator at 37 °C with 5% CO_2_. All transformed cells were tested for mycoplasma and were maintained in culture for up to 2 months. CX-4945 was made up as a 40 mM stock in DMSO (AdooQ Bioscience, Irvine, CA, USA). Cisplatin was obtained from the Minneapolis VA hospital pharmacy at a concentration of 1 mg/mL.

### 2.2. siRNA Transfections

Standard chemistry siRNAs were obtained from Dharmacon (ThermoFisher Scientific, Waltham, MA, USA). The siCK2α sense strand sequence is 5′-auacaacccaaacuccacauuu-3′ [25]. The CK2α’ sense strand sequence is 5′-auacagcccaaacuccacauuu-3′ [25]. The CK2α and CK2α’ siRNAs were cotransfected in a ratio of 3:1. The control siRNA (siControl) used was siNon-targeting #2 (Dharmacon; D-001810-02). Transfections of siRNA were performed on 60 mm plates with cells at 40–50% confluence using Dharmafect 1 and 2 reagents using 10 µL of Dharmafect and 20 nM siRNA total concentration [26].

### 2.3. Combination Treatments and Viability Assays

For siRNA/cisplatin experiments, cells were trypsinized from 60 mm plates after 24 h of transfection, and transferred into 96-well plates (Primaria, Thermo Fisher Scientific; Detroit-562 3500 cells; Fadu 2500 cells). Cisplatin was added at 48 h of transfection in a 2-fold dilution series from 20 to 0.62 µM. Viability assays were performed at 96 h of siRNA transfection and 48 h of cisplatin treatment. Success of transfection was judged by visual confirmation on 96-well plates that siCK2 transfected cells (without cisplatin) were 2–4-fold less confluent with predominantly apoptotic morphology compared to siControl transfected cells, which were not apoptotic. Calculation of IC50 values used siControl as the comparator control treatment. For CX-4945/cisplatin experiments, cells were plated into 96-well Primaria plates (Detroit-562 4000 cells; Fadu 3000 cells; UM-SCC-6 6000 cells; UM-SCC-47 6000 cells; 93-Vu-147T 6000 cells). CX-4945 was added in a 2-fold dilution series from 50 to 0.39 µM for Detroit-562 and Fadu and from 100 to 0.78 µM in UM-SCC-6, UM-SCC-47, and 93-Vu-147T. Cisplatin was added in a 2-fold dilution series from 80 to 0.62 µM. DMSO was added to cisplatin treated cells at the same concentration as used for CX-4945 single treatments to control for DMSO effects. Viability was measured at 72 h of CX-4945 and 48 h of cisplatin treatment. Calculation of IC50 values used untreated cells as the comparator control treatment. CellTiter 96^®^ Aqueous One or CellTiter-Blue^®^ Assays (Promega Corp., Fitchburg, WI, USA) were used to assess cell viability following treatments. Assays were performed according to the manufacturer instructions [27]. Absorbance was measured at 490 nm for Aqueous One and at 560 nm excitation/590 nm emission for CellTiter Blue using a Molecular Devices 5 plate reader with absorbance values for media alone subtracted from the experimental values.

### 2.4. Cell Treatments and Immunoblot Analysis

Detroit-562 and Fadu cells were transfected as described above. After 24 h of transfection, cisplatin was added to cells to a final concentration of 1 µM for an additional 24 h. For CX-4945/cisplatin treatments, CX-4945 was added to cells for a total of 48 h and cisplatin for a total of 24 h. The drug concentrations (µM) for CX-4945/cisplatin for each cell line were as follows: Detroit-562 1.5/5; Fadu 2.5/5; all others 5/5. Cell pellets were processed in radioimmunoprecipitation assay (RIPA) buffer, and 20 µg of each lysate was subjected to electrophoresis using TGX 5–15% midi gel system (BioRad, Hercules, CA, USA) and wet tank transfer to nitrocellulose membrane, as described [28]. After transfer, the membranes were fully dried, rehydrated in nano-pure water, and blocked for 30 min with 5% nonfat milk (Bio-Rad 170-6404) or 5% bovine serum albumin (Sigma A-9647) in Tris buffered saline (TBS, pH 7.4) with 0.1% Tween 20 (TBS-T) at room temperature. Antibodies were diluted into fresh blocking buffer according to the manufacturer’s recommendations, and the membranes processed as described [28]. Antibodies used: CK2α (A300-197A) and CK2α’ (A300-199A) from Bethyl Laboratories (Montgomery, TX, USA); CK2α’ (CSNK2A2) from ABclonal (A1616; Woburn, MA, USA); CK2β (sc-46666), NFκB p65 P-S529 (sc-101751), RB (sc-102), and actin (sc-1616) from Santa Cruz Biotechnology (Santa Cruz, CA, USA); NFκB p65 (6956), Bax (2772), Bak (121505), p21 (2947), PDCD4 (9535), p53 (48818), AKT-1 (2967), and AKT-1 P-S129 (13461) from Cell Signaling (Danvers, MA, USA). Proteins were detected by enhanced chemiluminescence using Pierce SuperSignal West Pico Plus and Dura substrates (Pierce 34580, 34076). Chemiluminescent signal was detected using the LiCor Odyssey Fc instrument, with quantitation performed using Image Studio 5.2.

### 2.5. Statistical Analysis

Table 2 includes descriptive statistics. PDCD4 immunoblot data was analyzed by the Mann–Whitney U test (2-sided). Viability curves and IC50 calculations were performed using GraphPad Prism 9. Synergy calculations for combination index were performed using Compusyn [29].

## 3. Results

### 3.1. CK2 Expression and Activity in HPV+ and HPV- HNSCC Cell Lines

**Figure 1:**
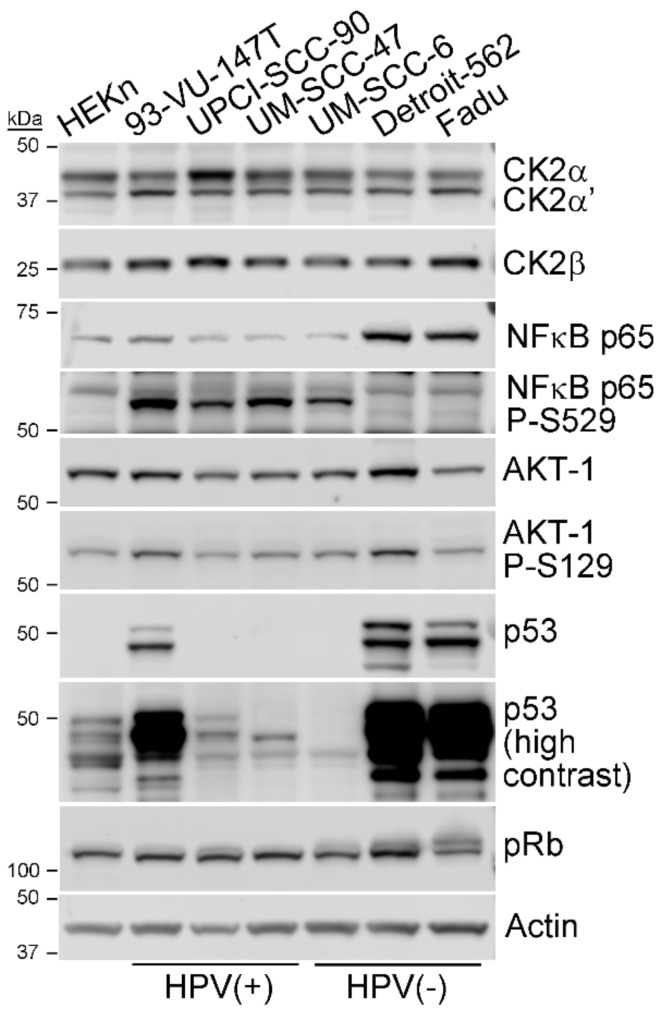
Expression of CK2 subunits and key markers in untransformed cells and HNSCC cell lines. Immunoblot analysis of cultured cell lines, as indicated above the blots. CK2α and CK2α’ antibodies were combined for simultaneous detection of these 2 proteins. Proteins detected are indicated on the right side of the blots. Molecular mass markers are indicated on the left side of the blots. Actin signal was used as the loading control.

We evaluated CK2 levels and surrogate activity in representative HPV(+) and HPV(-) cell lines. In order to evaluate CK2 subunit expression in HNSCC cell lines, we employed 3 HPV+ and 3 HPV- cell lines and a non-transformed human epithelial keratinocyte cell line (Table 1). Under standard growth conditions, CK2 subunit protein expression was examined by immunoblot analysis. The three subunits of CK2 were well expressed in all cell lines (Figure 1), and steady-state expression levels were 1.4–1.9-fold higher in HPV+ cell lines compared to HPV- (Table 2; Table S1). We also investigated markers of CK2 activity; specifically, phosphorylation of the CK2-specific sites on NFκB p65 (S529) and AKT-1 (S129) [30,31]. CK2 phosphorylation of NFκB p65 at S529 was dramatically higher in HPV(+) compared to HPV(−) cell lines (Figure 1, Table 2). In contrast, AKT-1 phosphorylation at the CK2 site was roughly equivalent in both types of HNSCC cells. Expression of p53 was detected in all cell lines, with much higher levels in the cells with mutant p53. Overall levels of pRb were similar in all cell lines, with higher molecular weight species evident in the HPV(−) cell lines.

### 3.2. HNSCC Cell Viability Following Cisplatin Treatment and Reduced CK2 Activity or Expression

**Figure 2:**
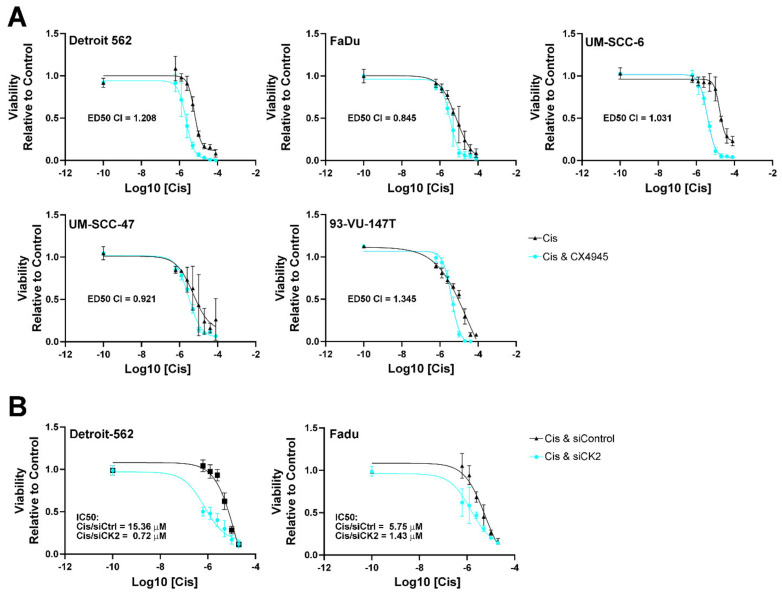
Viability curves for cisplatin treatment alone or combined with reduced CK2 activity or expression in HPV(+) and HPV(−) HNSCC. Cells were treated and viability measured using MTT-related assays as described in Materials and Methods. Log10 cisplatin dosing is indicated on the *X*-axis and viability relative to control is indicated on the *Y*-axis. (**A**) Cisplatin anchored analysis viability curves for cisplatin alone or combined with CX-4945. Cisplatin treatment alone is indicated by black triangles, and combined CX-4945 plus cisplatin treatment is indicated by blue circles. *N* = 4. IC50 values are shown in Table 3. Combination Index for 50% loss of viability is indicated on each curve (ED50 CI). (**B**) Viability curves for cisplatin treatment in siCK2 or siControl transfected cells. Cisplatin/siControl treatment is indicated by black squares (Detroit-562) and black triangles (Fadu), and cisplatin/siCK2 treatment is indicated by blue circles. *N* = 4. IC50 values are indicated on each curve. 95% CI for Detroit-562: Cis + siControl (8.99, 31.22 µM), Cis + siCK2 (0.43, 1.2 µM). 95% CI for FaDu: Cis + siControl (3.47, 10.37), Cis + siCK2 (0.77, 2.79 µM).

Given the widespread clinical use of cisplatin therapy for both HPV(+) and HPV(−) HNSCC cases and the general reliance of HNSCC cells on CK2 activity, we investigated the potential utility of combined CX-4945 treatment with cisplatin treatment [32,33]. Specifically, we evaluated the viability of HNSCC cells after cisplatin, CX-4945, and combined CX-4945 followed by cisplatin treatment. Adding CX-4945 to cisplatin treatment further reduced cell viability over cisplatin alone and significantly decreased the IC50 for cisplatin for all cell lines but UM-SCC-47. The fold decrease with the addition of CX-4945 ranged from 1.7 to 7.9 (Table 3 and Figure 2A). In reverse analysis, when we determined the effect of cisplatin addition to CX-4945, the effect was to slightly reduce the IC50 for CX-4945, from 1.1 to 2.0-fold (Table 3). These results suggest that under the treatment conditions that we used, CX-4945 treatment was the dominant effect reducing cell viability in both HPV(+) and HPV(−) cells. We analyzed this data for synergy and found that CX-4945 and cisplatin combined treatment was synergistic in the HPV(+) cell line UM-SCC-47 and the HPV(−) cell line Fadu (Figure 2A). CX-4945 and cisplatin combined treatment was additive in the HPV(−) cell line SCC-6 (Figure 2A).

We also examined the viability of Detroit-562 and Fadu cells following siRNA-mediated CK2 downregulation and cisplatin treatment. Cells were transfected with a constant amount of either CK2-targeting or control siRNAs and treated 2 days later with cisplatin for an additional 48 h. Similar to the combined effects of CX-4945 with cisplatin, the viability curve was shifted to the left with significantly reduced IC50s for cisplatin when CK2 expression was inhibited by 4 to 21-fold (Figure 2B).

### 3.3. Signaling Response of HNSCC cells to CK2 Targeting Using CX-4945 or siRNA and Cisplatin Treatment

**Figure 3:**
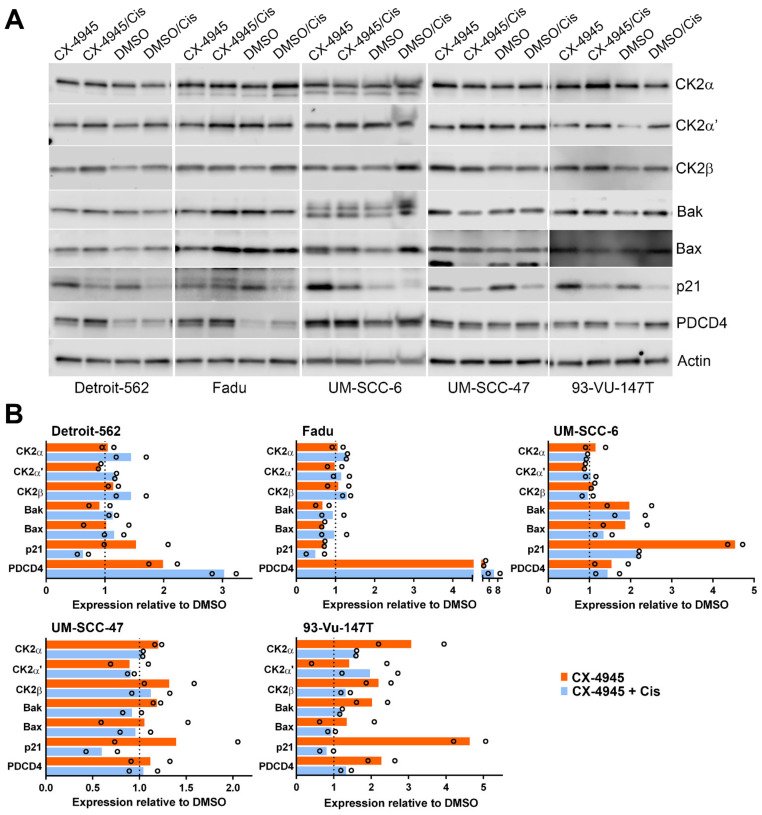
Immunoblot analysis of various signals following CX-4945 treatment alone or combined with cisplatin in HNSCC. Cells were treated and protein expression measured using immunoblot assays as described under Materials and Methods. The drug concentrations (µM) for CX-4945/cisplatin for each cell line were as follows: Detroit-562 1.5/5; Fadu 2.5/5; all others 5/5. (**A**) Representative blots from immunoblot analysis of HNSCC cells following CX-4945 treatment (48 h) with and without cisplatin (24 h). Proteins detected are indicated on the right side of the blots. Actin signal was used as the loading control. (**B**) Charts representing quantitation of protein signals relative to DMSO control treatment. Orange = CX-4945 treatment alone. Blue = CX-4945 and cisplatin treatment combined. Black open circles represent each data point from 2 biological replicate immunoblots.

We further examined treatment response to CX-4945 treatment alone and in combination with cisplatin in 3 HPV(−) and 2 HPV(+) cell lines by immunoblot. We examined the cells, using sequential treatment, with CX-4945 treatment for 48 h and cisplatin treatment for 24 h. This relatively short treatment regimen was chosen to examine CX-4945-induced changes in HPV(−) and HPV(+) HNSCC cells prior to the full cascade of death signaling. Overall, CX-4945 treatment alone or combined with cisplatin caused no consistent change in the proapoptotic proteins Bak and Bax; although some induction of Bak and Bax was observed in UM-SCC-6 and 93-Vu-147T cells (Figure 3). CX-4945 caused induction of the cyclin dependent kinase inhibitor p21 from 1.4- to 4.6-fold in four of the cell lines; the presence of cisplatin generally reversed p21 induction in combined treatment. Cisplatin treatment alone caused reduction of p21 below 60% of control cells (Figure 3). Levels of the tumor suppressor programmed cell death 4 protein (PDCD4) were elevated 1.5- to 5-fold following CX-4945 inhibition in four of the five cell lines, with further increase upon cisplatin addition in two of these cell lines. Addition of cisplatin to CX-4945 treatment slightly increased CK2 protein levels in four of the five cell lines.

**Figure 4:**
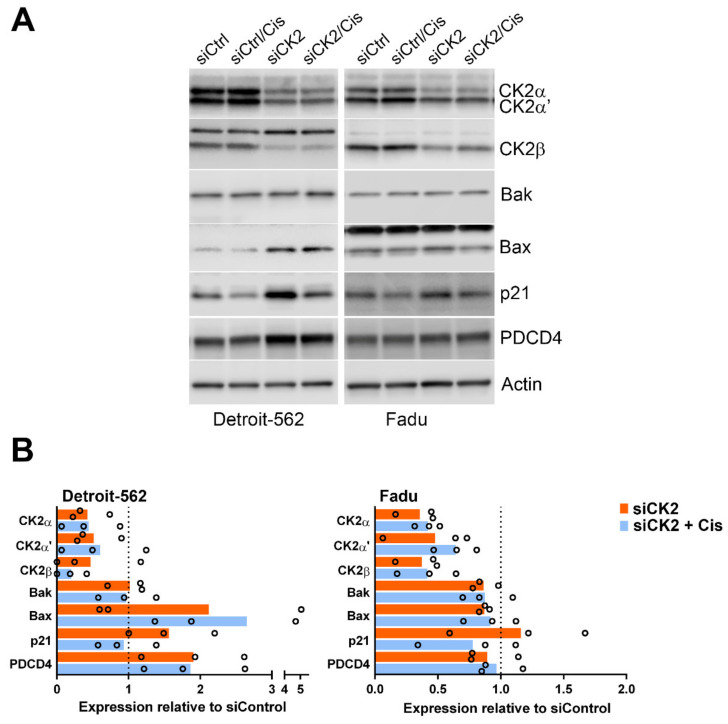
Immunoblot analysis of various signals following CK2 downregulation alone or combined with cisplatin in HNSCC. Cells were treated and protein expression measured using immunoblot assays as described under Materials and Methods. (**A**) Immunoblot analysis of Detroit-562 and Fadu cells following siRNA transfection (48 h) with and without cisplatin (24 h) carried out as described under Materials and Methods. CK2α and CK2α’ antibodies were combined for simultaneous detection of these 2 proteins. Proteins detected are indicated on the right side of the blots. Actin signal was used as the loading control. (**B**) Charts representing quantitation of protein signals relative to si-Control treatment. Orange = siCK2. Blue = siCK2 and cisplatin treatment combined. Black open circles represent each data point from 3 biological replicate experiments. siCtrl = siRNA for non-targeting control.

Detroit-562 and Fadu cells were also transfected with a siRNA cocktail to specifically knockdown CK2α and CK2α’ protein expression. Reduced expression of the CK2 subunits was confirmed (Figure 4). Many of the observations from CX-4945 treatments were replicated for Detroit-562, including induction of p21 and PDCD4 by loss of CK2 (Figure 4). One difference was that Bax levels increased following downregulation of CK2 combined with cisplatin as opposed to CX-4945 plus cisplatin treatment. The response of Fadu cells to siRNA-mediated CK2 loss with and without cisplatin was very similar to that for the use of CX-4945, with the exception that CK2 downregulation did not induce PDCD4. CK2 knockdown in these two cell lines only slightly induced PDCD4 mRNA (Detroit-562: 1.36 ± 0.49; Fadu: 1.09 ± 0.07).

**Figure 5:**
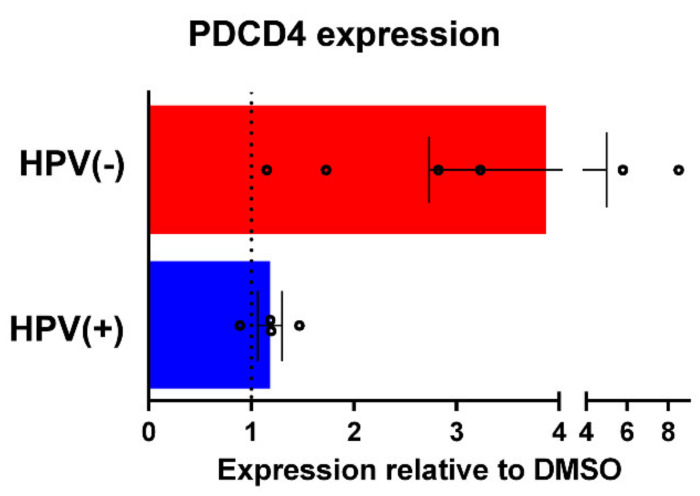
Comparison of PDCD4 induction following combined CX-4945 and cisplatin treatment in HPV(−) vs. HPV(+) HNSCC cells. The mean and SEM of PDCD4 immunoblot signals relative to DMSO control treatment is depicted. Red = HPV(−). Blue = HPV(+). Circles represent each data point.

Finally, we grouped the HPV(−) versus HPV(+) cell line immunoblot data together to evaluate any differences between HNSCC cells according to viral status. One striking difference was the large increase in PDCD4 level in HPV(−) cells compared to HPV(+) (Figure 5, *p* = 0.07) following combined CX-4945 and cisplatin treatment. No other notable differences were observed.

## 4. Discussion

Given the rise in HPV-related HNSCC, we undertook this examination of CK2 level in relation to HPV status and the effect of CK2 targeting as a cotreatment with cisplatin. We observed higher steady-state CK2 protein levels and kinase activity directed to NFκB p65 in HPV(+) cells relative to HPV(−). It appears that under conditions of HPV infection, CK2 protein levels are elevated by a currently unknown mechanism to meet the demands of viral replication. CK2 regulates HPV proteins such as E1 and E7 involved in the papillomavirus lifecycle. The HPV E7 oncoprotein promotes immortalization and transformation in infected cells through inactivation of pRb and related pathway proteins [34]. Previous studies have demonstrated key regulation of E7 function by CK2 [18,19,20,35]. For example, the phosphorylation of E7 by CK2 is essential to promote Rb-related p130 degradation and cell cycle S-phase entry [36,37]. CK2α is required for HPV DNA replication by regulating the stability and nuclear retention of E1, and CK2 has been proposed as a promising target for the development of antiviral drugs [21,38].

Cervical cancer is almost entirely associated with positive HPV status. In other work, it was shown that targeting of CK2 activity using an investigational peptide inhibitor is effective in treating cervical cancer [39,40]. This CK2 inhibitor, CIGB-300, was identified in a screen of peptides, which bind and block phosphorylation of an HPV16 E7 fusion protein [41]. Combinatorial use of CIGB-300 with cisplatin demonstrated a good synergy and/or additivity profile against a cervical cancer cell line, and improved survival in mouse xenograft studies [42]. These observations are analogous to those described here on the combinatorial treatment of HNSCC with CK2 inhibitor CX-4945 and cisplatin.

Treatment of malignant cells with cisplatin and/or radiation causes nuclear DNA damage and redox stress, mitochondrial DNA damage, and mitochondrial outer membrane permeabilization [43,44,45]. Over time, adaptation to cisplatin in malignant cells results in therapeutic failure and tumor recurrence in patients. We have previously shown in prostate cancer that CK2 inhibition has a negative impact on mitochondrial health through decreased membrane potential and Ca^2+^ flux [27,46]. CK2 has significant influence on numerous DNA repair and other pathways activated by radiation and cisplatin [27,47,48,49]; loss of CK2 improves sensitivity to cisplatin or radiation in numerous cancers, including head and neck cancer [42,50,51,52,53,54,55,56,57,58,59,60,61,62]. CX-4945 blocks DNA repair after cisplatin or gemcitabine treatment [51,63], and next generation platinum Pt(IV) prodrugs conjugated with CX-4945 have shown efficacy in other cancer types [64]. In a pilot study, metastatic HNSCC lesions were successfully treated in nude mice using a combination of cisplatin and tumor directed nanocapsules containing RNAi oligonucleotides against CK2 [65]. Together, these data suggest that incorporating CK2 blockade could improve or prolong the response to cisplatin therapy by hindering DNA repair and influencing mitochondrial health in both HPV(+) and HPV(−) HNSCC [66].

A key downstream target of CK2 activity in HNSCC is NFκB. The NFκB complex plays broad roles promoting proliferative and inflammatory pathways, and is aberrantly activated in numerous cancers, including HNSCC [65,67]. CK2 modulates IKKβ and IκBα phospho-states and degradation, promotes IKK-mediated phosphorylation of NFκB p65 at S536, and directly activates p65 by phosphorylation at S529 [65]. We have previously shown that CK2 knockdown modulates NFκB activity and sensitizes HPV(−) HNSCC cells to cisplatin [50]. Our data here show a new link between high CK2 levels and CK2-activated NFκB, but not CK2-activated AKT-1, in HPV(+) HNSCC cells. Transcriptome analysis demonstrated that NFκB and death signaling pathways differed according to HPV status [68]. Future studies could further investigate CK2 signaling in HPV(+) HNSCC in relation to NFκB activity and induction of cell death.

Loss of tumor suppressor gene or protein expression is proposed as part of HNSCC oncogenesis [14]. It was previously shown in HNSCC that molecular downregulation or kinase inhibition of CK2 enhanced levels of the tumor suppressor TAp73 and inhibited expression of cancer stem cell genes and side population [50,69]. The tumor suppressor PDCD4 protects cells from neoplastic transformation, exhibits reduced expression levels in malignant compared to non-transformed cells, and functions to inhibit protein translation [70,71,72]. PDCD4 protein expression is regulated by multiple microRNAs in HNSCC, and loss of PDCD4 renders cancer cells more resistant to cisplatin [73,74,75]. PDCD4 interacts with CK2 in the nucleus and is a predicted CK2 substrate [76,77]. Our results showed for the first time that PDCD4 protein levels were strongly induced in HNSCC by CX-4945 treatment, especially in the p53 mutant cell lines Detroit-562, Fadu and 93-Vu-147T. CK2 knockdown using siRNA inexplicably raised PDCD4 in Detroit-562 but not Fadu cells. At present it is unclear what molecular pathways or genetic characteristics influence PDCD4 abundance following interruption of CK2 activity or expression.

CX-4945 treatment or CK2 downregulation also strongly induced tumor suppressor p21 levels, a cyclin dependent kinase inhibitor promoting cell cycle arrest [78]. Induction of p21 was shown to occur when cisplatin-resistant HPV(−) HNSCC cells were resensitized using palbociclib and JQ1 [79]. Here we observed increased p21 protein after CX-4945 treatment in both HPV(+) and HPV(−) HNSCC. Elevation of p21 is due to CK2 knockdown in two of two cell lines and CX-4945 treatment in four of five cell lines. We noted a different CX-4945 related p21 molecular response in the Fadu cell line. The heterogeneity of malignancies observed in patients and the corresponding cancer cell lines has emerged as a fundamental tenet of cancer studies over the past many years. It is not unusual that the HNSCC cell lines we employed in this study occasionally showed different responses to experimental manipulation, and in fact this is to be expected. Thus, we conclude that in most HNSCC cell lines, blocking CK2 activity or expression allows elevation of p21. The recovery of PDCD4 protein levels and the induction of p21 and TAp73 may represent potential mechanisms by which reducing CK2 activity or expression levels improves cisplatin sensitivity in HNSCC cells.

Ongoing clinical trials continue to support the potential efficacy of CX-4945 in different therapeutic contexts in a range of malignancies (clinicaltrials.gov). The small molecule inhibitor CX-4945 is not solely selective for CK2 activity, due to some off-target inhibition of and/or interaction with several other kinases [80,81]. In light of the off-target effects of CX-4945, it is important to note that data from studies using CX-4945 significantly overlap with data generated in vivo and in vitro by knockdown of CK2 gene expression, as shown here and in numerous other cancer types [82,83,84,85,86,87,88,89,90,91,92]. A recent summary of an original research publication states that the development of a new highly selective chemical probe for CK2 “challenges the broad cancer essentiality of CK2” [93]. The chemical probe SGC-CK2-1 did not cause notable loss of proliferation in greater than 90% of more than 140 cancer cell lines, which was a surprising finding [94]. However, there was no discussion of published data on the effects of CK2 knockdown using RNA interference and CRISPR/Cas9 techniques. The strong foundation of evidence that molecular downregulation of CK2 protein levels induces loss of cancer cell viability and tumor cell death should not be discounted. Further research using SGC-CK2-1 will hopefully shed some light on this observed discrepancy.

**Figure 6:**
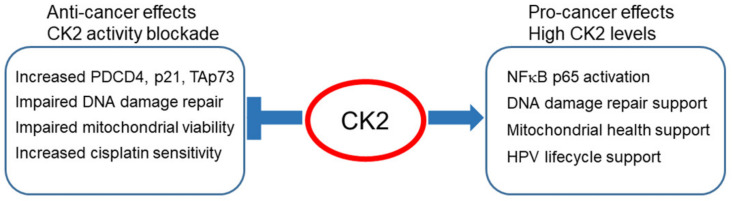
Impact of CK2 level and activity in HNSCC. A summary of results presented here and previously published from this group and others is depicted. Increased cisplatin sensitivity following CX-4945 or CK2 downregulation is observed in both HPV(+) and HPV(−) HNSCC cells.

Current therapy of HNSCC relies on surgery, radiation, and/or chemotherapy, typically cisplatin. A significant number of patients, especially those with HPV(−) disease, suffer poor outcomes after therapy; while patients who experience long-term benefits also develop persistent cisplatin-related toxicity. This study shows significantly reduced IC50 values for cisplatin in multiple HPV(+) and HPV(−) HNSCC cell lines under combined treatment with CX-4945 or after CK2 knockdown, suggesting a path toward a therapy strategy allowing reduced cisplatin usage. HNSCC is the 6th most common cancer in the world, and new treatment modalities that can improve outcomes and reduce toxicities are needed [14,95,96]. We summarized in Figure 6 the results presented here and in other published works related to loss of CK2 activity and potential pathways of achieving improved HNSCC response.

### Future Directions and Limitations

Extended work could examine cisplatin treatment as the first drug since the CX-4945 treatment dominated the loss of viability in these studies, as suggested by the data in Table 3. The possible off-target effects of CX-4945 are well documented, and further investigation into the role of CK2 downregulation through the use of other inhibitors and/or CK2 molecular downregulation and would define the contribution of off-target kinases.

## 5. Conclusions

To our knowledge, this is the first report evaluating CK2 expression level in relation to HPV status in HNSCC cells. Treatment with CX-4945 resulted in significantly decreased IC50 values for combined treatment with cisplatin in both HPV(+) and HPV(−) HNSCC cell lines; synergy was observed in a subset of cell lines. Phase 1 and 2 clinical trials demonstrated that the oral CK2 inhibitor CX-4945 is safe for use in cancer patients, slowing disease progression and extending treatment benefit for some patients with advanced solid tumor cancers. Our results suggest that HNSCC patients could benefit from further investigation into the incorporation of CX-4945 into treatment strategies. In addition, the function of CK2 in HPV biology as it pertains to HNSCC requires further study given the emergence of HPV(+) HNSCC.

## Figures and Tables

**Table 1:** Characteristics of head and neck cell lines.

Cell Lines	Tissue Origin	Age (yr)	Sex	HPV Status	*CDKN2A* Status ^1^	*TP53* Status ^1^
HEKn ^2^	Foreskin	<1	Male	-	ND ^3^	ND ^3^
Detroit 562	Pharynx (metastatic pleural effusion)	ND ^3^	Female	-	Homozygous mutant	Homozygous mutant
Fadu	Hypopharynx	56	Male	-	Homozygous mutant	Heterozygous mutant, both alleles
UM-SCC-6	Base of tongue	32	Male	-	Homozygous deletion	Wild-type
UM-SCC-47	Lateral tongue	53	Male	+	Wild-type	Wild-type
UPCI-SCC-90	Base of tongue	46	Male	+	Wild-type	Wild-type
VU-SCC-147T (93-Vu-147T)	Floor of mouth	58	Male	+	Wild-type	Mutant

^1^ As determined from: ATCC.org; Expasy.org; broadinstitute.org/ccle; cancer.sanger.ac.uk/cosmic. ^2^ Primary human epidermal keratinocytes from neonatal foreskin. ^3^ ND—not determined or known.

**Table 2:** Comparison of CK2 abundance and CK2-related activity immunoblot signals between HPV(+) and HPV(−).

Status	CK2α	CK2α’	CK2β	NFκB p65 total	NFκB p65 P-S529	p65 P-S529/Total	AKT-1 Total	AKT-1 P-S129	AKT-1 P-S129/Total
HPV+	1.00 ± 0.49	0.62 ± 0.14	2.92 ± 0.86	0.02 ± 0.01	2.18 ± 0.59	145.97 ± 44.79	0.56 ± 0.04	1.12 ±0.23	2.02 ± 0.31
HPV-	0.55 ± 0.10	0.423 ± 0.02	1.96 ± 0.23	0.03 ± 0.02	0.33 ± 0.51	35.52 ± 67.14	0.51 ± 0.19	1.10 ± 0.56	2.07 ± 0.29
HPV+/HPV-	1.82	1.450	1.49	0.48	6.65	3.69	1.08	1.02	0.97

Mean signal, expressed relative to actin, for 3 each HPV(+) and HPV(−) cell lines ± standard deviation.

**Table 3:** IC50 calculations from single and combined treatments.

	IC50 (µM)		IC50 (µM)	
	Cisplatin Anchored Analysis		CX-4945 Anchored Analysis	
Cell Line	Cisplatin	Cisplatin with CX-4945	CX-4945	CX-4945 with Cisplatin
Detroit 562	8.03	2.42	1.92	1.46
	(5.49, 11.97)	(1.72, 3.46)	(1.51, 2.45)	(1.03, 2.09)
Fadu	7.35	3.41	4.36	2.13
	(5.06, 10.82)	(2.51, 4.67)	(2.88, 6.78)	(1.54, 2.98)
UM-SCC-6	31.37	3.95	5.58	4.94
	(18.99, 55.56)	(3.00, 5.24)	(4.24, 7.40)	(3.75, 6.56)
UM-SCC-47	5.12	2.94	4.77	3.67
	(2.20, 12.19)	(2.39, 3.63)	(3.82, 5.99)	(2.98, 4.53)
93-Vu-147T	9.52	4.05	5.7	5.07
	(6.37, 14.23)	(3.18, 5.19)	(4.43, 7.37)	(3.98, 6.48)

*N* = 4. 95% confidence intervals in parentheses.

## Data Availability

The data presented in this study are available on request from the corresponding author.

## Supplementary Materials

The following are available online: https://www.mdpi.com/article/10.3390/biomedicines9050571/s1. Table S1, Quantitation of HNSCC cell line immunoblot signals.
