# Supplementary material for: CX-4945 and siRNA-Mediated Knockdown of CK2 Improves Cisplatin Response in HPV(+) and HPV(−) HNSCC Cell Lines
Source: Biomedicines. 2021 May 18;9(5):571. doi: 10.3390/biomedicines9050571 (PMC8158385; doi:10.3390/biomedicines9050571)
Supplement: Supplementary file 1 [file biomedicines-09-00571-s001.zip › Table S1.pdf]

Table S1. Quantitation of HNSCC cell line immunoblot signals[1.]

Cell Line	CK2α	CK2α'	CK2β	NFκB p65	NFκB p65 P-S529	AKT-1	AKT-1 P-S129	p53	pRb
HEKn	0.988	0.572	2.478	0.017	.0101	0.621	0.775	3.993	13.060
Detroit 562	0.508	0.431	1.839	0.053	0.046	0.716	1.723	47.621	24.980
Fadu	0.474	0.437	2.226	0.040	0.026	0.349	0.652	29.944	16.256
UM-SCC-6	0.668	0.408	1.828	0.008	0.912	0.472	0.917	0.679	13.593
UM-SCC-47	0.779	0.459	2.127	0.009	1.777	0.513	1.076	1.171	18.340
UPCI-SCC-90	1.562	0.717	3.842	0.016	1.905	0.551	0.923	2.529	25.117
93-VU-147T	0.670	0.674	2.804	0.024	2.864	0.620	1.375	20.679	15.749
